# Parents' intention toward early marriage of their adolescent girls in eastern Ethiopia: A community-based cross-sectional study from a social norms perspective

**DOI:** 10.3389/fgwh.2022.911648

**Published:** 2022-10-05

**Authors:** Dureti Abdurahman, Nega Assefa, Yemane Berhane

**Affiliations:** ^1^Department of Public Health, College of Health and Medical Sciences, Haramaya University, Harar, Ethiopia; ^2^Department of Epidemiology and Biostatistics, Addis Continental Institute of Public Health, Addis Ababa, Ethiopia

**Keywords:** social norms, intention, unmarried girls, parents, Ethiopia

## Abstract

**Background:**

Strong social norms around early marriage put pressure on parents to marry off their adolescent girls at an early age. Early marriage is widely practiced in many parts of Ethiopia. However, early marriage studies rarely address the role of social norms. This study aims to examine the role of social norms on parents' intention to marry off adolescent girls early in Eastern Ethiopia.

**Method:**

A community-based cross-sectional study was conducted from September to November 2019. A multistage sampling procedure was applied to select the study participants. Factors related to parents' intention for early marriage were assessed through the lens of the Theory of Planned Behavior (TPB). Adjusted Prevalence Ratio (PR) was calculated using a log-binomial model to identify factors associated with intention toward early marriage.

**Results:**

A total of 859 mothers and 859 fathers of unmarried girls were included in the study. The prevalence of intention to marry off their daughter early among mothers was 39.70% (95% CI = 36.4–43.0%) and 43.54% (95%CI = 40.2–47.1%) among fathers of adolescent girls [chi2(1) = 41.8; *P* < 0.001]. The intention for an early marriage was higher among mothers and fathers with favorable attitude towards early marriage, in those who believe that most people in their reference group conform to early marriage norms (empirical expectation), in those who believe that most people in their reference group expect them to conform to early marriage norms (normative expectation) and among fathers who did not know the legal age of marriage for adolescent girls and those who did not know the health consequences of early marriage. The prevalence of intention toward early marriage was lower among mothers (APR = 0.49; 95% CI: 0.36–0.65) and fathers (APR = 0.62; 95% CI: 0.49–0.78) in urban areas; and among mothers (APR = 0.41; 95% CI: 0.27–0.62) and fathers (APR = 0.50; 95% CI: 0.38–0.67) with higher educational level.

**Conclusion:**

The social norms that promote intention for early marriage are still high among parents, especially among those living in rural areas and uneducated. Hence, interventions that aim to reduce early marriage need to address locally relevant social norms.

## Introduction

Marriage practices are largely dominated by culture and tradition even in the presence of secular laws governing marriage (1). In low-income countries, early marriage is not uncommon, especially among girls, who then take the role of being a wife and mother at an early age before they gain physical and mental maturity (2, 3). Early marriage is a marital union that occurs when one or both parties are younger than 18 years of age (4, 5). The practice has been recognized as a global human rights violation under Article 16 of the 1948 Universal Declaration of Human Rights (6).

Early marriage is overwhelmingly prevalent across countries, cultures, and religions. Recently more than 700 million under-aged girls married globally, and of this, more than half occurred in South Asia, Latin America, and Sub-Saharan Africa (7). Though the age of marriage is gradually increasing, early marriage is still a common problem in Sub-Saharan Africa; the region has the highest rate of early marriage in the world (8, 9). Early marriage is also a common practice among Ethiopian women. The prevalence of early marriage among reproductive-age women ranges from 26% in Addis Ababa to 87% in Amhara's eastern region (10). Furthermore, the national prevalence of early marriage was 58 % in 2016 (11).

The practice of early marriage has severe consequences on the health, psychological, physiological, and socio-economic wellbeing of young girls and their infants (12, 13). Girls who marry early are often denied a range of human rights, prevented from studying further, face serious health risks from early and multiple pregnancies, and suffer sexual and domestic violence. It also has implications on the overall development and wellbeing of society as a whole (14, 15).

Although early marriage has negative consequences, various studies have shown the persistence of support for early marriage by parents because of a variety of factors (16, 17). In patriarchal societies, such as in Ethiopia, parents are the primary decision-makers on marriage, and on behalf of their daughters, parents decide when and whom to marry (18). The most commonly cited reasons in favor of early marriage include the intention to improve the economic status of the family (19), the parent's desire to become grandparents, to strengthen ties between the marrying families, “protection” from out-of-wedlock pregnancies, family honor, and customary or religious laws that support the practice (20–23). Parents also believe they need to follow their reference group or local behavioral rules to conform to socially acceptable norms (24).

In Ethiopia, early marriage is often arranged or negotiated by family members (25). Parental influence over the early marriage of girls is well-documented in qualitative research, but little quantitative work has been done about the influence of social norms on early marriage practices. Similarly, in many other low-income countries the link between early marriage and social norms has not been studied widely (24). Couples share similar experiences after marriage as a result of their shared lives and they never parent in isolation, more so in a social and cultural context where attitudes are transmitted through spousal communication. Since parenting, parents, and cultures are inextricably linked, their attitudes are likely to influence one another, and they share common cultural beliefs and values toward their children (26). However, background differences or events experienced throughout their lives can lead to disparities in attitudes and perceptions. Parents' attitudes may influence their children independently if their attitudes and perceptions do not overlap (27). In addition, patriarchal norms continue to perpetuate gender inequalities in Ethiopia, where women are generally considered subordinate to their husbands, have less power in relationships due to their economic, political, and sociocultural status, and may not be able to decide on the sexual and reproductive health matters of the household (28). Moreover, the more gendered social experiences are, the more the couples' attitudes and perceptions will differ. We addressed both parents from the household for these reasons. Therefore, this study aims to examine the association between parents' intention to marry off their adolescent girls early and social norms around marriage in Eastern Ethiopia.

## Methods and materials

### Study setting and design

A community-based cross-sectional study was conducted in Kersa HDSS, Eastern Hararghe zone of Oromia regional state, in the eastern part of Ethiopia. The fieldwork was done from September to November 2019. The Kersa HDSS site, where the study was conducted, consists of 21 rural and three urban kebeles (the smallest administrative unit in Ethiopia). It has 18 elementary, two secondary, one college preparatory, and two religious schools. Kersa HDSS also has 6 health centers, and 19 health posts (29). In each kebele, there are two Health Extension Workers (HEWs) providing health promotion services. The HDSS has a total of 26,061 households, of which 5,207 had at least one unmarried adolescent girl aged 13–17 (30). The population is predominantly Muslim and lives on subsistence farming although some farmers cultivate cash crops. The Ethiopian society is patriarchal and traditional values dominate common social norms (31).

### Population and sampling technique

#### Population

The study population included both parents (either biological father/mother, female/male guardians or primary caretakers of the adolescents) of unmarried adolescent girls aged 13–17 and permanent residents of the study area. Parents who were critically ill and physically or mentally disabled at the time of data collection were excluded from the study.

#### Sample size determination

The sample size was determined using the double population proportion formula by assuming 50% of parents' early marriage intention, 95% CI, and a 5% margin of error. To maximize the sample size, a 20% non-response rate and a design effect of 2 were used; the final sample size for this study was 859. Since both parents were interviewed from a household the sample size was doubled, i.e., 1,718 (859 mothers and 859 fathers). The sample size for factors associated with intention of early marriage was calculated using Open Epi online with a two-sided confidence level (1-alpha) of 95% and power of 80% data (32). Accordingly, the minimum sample size was 108. However, the above sample size of 859 was used.

#### Sampling procedure

A multistage cluster sampling procedure was used to select study participants. First, we classified the rural kebeles into three strata (high land, middle land, and low land) and the urban kebele as one cluster. In the second stage, two kebeles from each of the three rural strata and two kebeles from the urban kebeles (eight clusters in total) were randomly selected. Then, the total sample size was allocated for each selected kebele proportional to the number of their target population. The list of households found in the selected kebeles with at least one unmarried adolescent girl was obtained from the Kersa HDSS. Then, households were drawn proportional to the size of the kebele population using a simple random sampling procedure based on the sampling frame obtained from KDSS. Both parents were interviewed from each selected household.

### Data collection

Data were collected face-to-face using a structured pre-tested questionnaire by trained and experienced field workers who were able to fluently speak the local language (Affan Oromo). The questionnaire was developed as described by Mackie et al. (33), to identify prevailing social norms in the study context and following TPB. In addition, sociodemographic and economic characteristics, the mothers' and fathers' knowledge of the legal age of marriage, legal and health consequences of early marriage, and access to mass media were collected using a household questionnaire. The questionnaire was originally developed in English and translated into Afan Oromo (the local language), then back to English to check the consistency. We pretested the study procedure and tools in a similar setting outside of our study area to test the appropriateness and clarity of the language used, the flow of questions, and the understanding of the questions by potential respondents. Based on the feedback obtained during the pre-test, some questions and the translation were refined. Before data collection, training was given to data collectors and field supervisors on survey procedures, study tools, and related issues by the researcher for 3 days. Data collectors scheduled interviews at a convenient time for the interviewees. Up to three visits were made if a respondent was not available on the first visit. The field supervisors closely supervised the data collection process moving home to home with data collectors and also checked each filled questionnaire for completeness on a daily basis. To ensure the confidentiality of their responses, data collectors interviewed eligible participants in a private setting. Moreover, to avoid the interference of spouses in a household, husbands and wives were interviewed separately.

### Variables and measurements

#### Outcome variable

In this study, parents' intention toward early marriage was the outcome variable. The intention was defined as the readiness of the mother or father to marry off their daughters before 18 years of age if a suitor comes forward (34). The intention was determined through a single question with a four-point Likert scale response: (1) “Disagree”, (2) “Somewhat disagree”, (3) “Somewhat agree”, and (4) “Agree”. For the analysis, we classified the responses into two categories: Not certainly agreed category (Disagree/somewhat disagree/Somewhat agree) and certainly agreed category (Agreed). This was further dichotomized and coded as “0” for not certainly agreed with intention and “1” for certainly agreed with intention”.

#### Explanatory variables

The explanatory variables were assessed using questionnaires adapted from previous literature with four-point Likert scale measurement tools (35, 36). We looked at questions across five domains: attitude toward early marriage (four items), the empirical expectation of early marriage (four items), the normative expectation of early marriage (five items), sanctions related to early marriage practices (two items), and whether early marriage is typical/commonly practiced among parent reference groups (one item). The detail of the questionnaires are attached in Supplementary Table 1.

The Theory of Planned Behavior (TPB) is a cognitive theory by Azjen (37) that unpacks an individual's decision to engage in a specific behavior. The theory explains the various factors that can influence an individual's behavior resulting from intentions, which in turn are influenced by attitudes, subjective norms, and perceived behavioral control. The more favorable the three factors are, the more likely the intention of the individual to be engaged in the particular action. It indicates that an individual's intentions are the best predictor of the person's behavior (38).

##### Attitude

It was defined as the tendency of mothers and fathers to judge and respond in the form of beliefs and feelings toward early marriage. It was measured by 4 items using a four-point Likert scale which were; “I think girls should get married before age 18”, “In my opinion getting married before 18 years is beneficial”, “If a girl did not marry early, she would not be marriageable”, and “An older girl will not find a good husband”. The responses ranged from ‘Disagree' (1) to Agree (4) and the total attitude scores ranged from 4–16. A dichotomous variable was created by summing all the items' scores using a mean split. Scores below the mean are taken as (an unfavorable attitude toward early marriage and a favorable attitude toward early marriage) otherwise.

##### Social norm

Social norm has two components: a rule that people follow because they believe that others do the same (empirical expectations), and believe others think they should do (normative expectation) (35).

##### Empirical expectation

It was measured by four items using a four-point Likert scale response ranging from 1 for “Disagree” to 4 for “Agree”. It included “Most of the girls in my community marry before the age of 18”, “Most parents in this community marry off their daughters early (<18)”, “All my neighbors marry off their daughters as soon as they think their daughters reach puberty”, and “Parents marry off their daughters early (<18 years) because they believe other parents do the same”. The total empirical expectation score ranged from 4–16 which was obtained by summing the scores of all items, and the mean was used to dichotomize the variable (36, 39). Those below the mean score were categorized as not agreed on empirical expectation toward early marriage and, the mean score and above were categorized as agreed on empirical expectation toward early marriage.

##### Normative expectation

Five items were used to measure the presence of normative expectations toward early marriage. It included: “Most of my friends think that girls should get married before age 18”, “Parents/father and mother/ expect adolescent girls to get married before the age of 18 years”, “Community elders expect parents to marry off their girls before the age of 18 years,” “Religious leaders expect parents to marry off their daughters before the age of 18 years”, and “Others community members expect parents to marry off their daughters before the age of 18 years”. The total normative expectation score ranged from 5–20 which was obtained by summing all items scores. Mean split was used to dichotomize and categorized as: not agreed on normative expectation toward early marriage if the mean was less, and agreed on normative expectation toward early marriage, if otherwise.

##### Sanctions

Sanctions were defined as social punishment or rewards (social consequences) that served to control early marriage-related actions (40). It was measured using two questions: “Parents would look down on adolescent girls if they get pregnant before they get married”, and “Parents who marry off their daughters before 18 years of age are recognized in the community as good parents”. Using a four-point Likert scale response ranging from “Disagree” (1) to “Agree” (4), the total index score was constructed by summing all items' scores. The mean split was used to dichotomize those below the mean score as not agreed on the presence of sanction toward early marriage, and as agreed on the presence of sanction toward early marriage, if otherwise.

##### Wealth index

Principal component analysis (PCA) was used to calculate household wealth. Three components were extracted based on factor loadings. Each household was categorized based on their score (lowest, medium, and highest) to represent poor, medium, and rich households respectively.

##### Knowledge of the legal age of marriage

The respondents were asked if they knew the legal age of marriage for girls, with a possible response option of Yes/No. Those who responded “Yes” were further asked to mention the legal age for marriage. Those who mentioned the legal age as “18 and above” were classified as respondents who “know the legal age of marriage.” Respondents who mentioned the legal age of marriage as “ <18 years” were classified as those who did “not know the legal age of marriage”.

##### Knowledge of legal consequences

The respondents were asked if they knew the legal consequences/punishment for marriage <18 years with a response option of Yes/No. Those responding “Yes” were further asked to mention the type of legal consequences/punishment for marriage <18 years from a listed choice.

##### Knowledge of health consequences of early marriage

The respondents were asked whether they knew of the health consequences of early marriage with a response option of Yes/No. Those responding with “Yes” were further asked to mention the consequences of early marriage from a listed choice.

### Data management and analysis

The questionnaires were double entered and cleaned using EpiData Version 3.1 and then exported to STATA 14 statistical software for analysis. Complete case analysis was used to handle the missing data since the missing values of each variable were less than one percent and considered missing completely at random. Percentages and their 95% confidence intervals were calculated for categorical variables and means were calculated for continuous variables. We examined the associations between intention and each independent variable by calculating the prevalence ratio (PR) using a log-binomial regression model which is preferable for the outcome variable with high prevalence (41). After checking for multicollinearity by examining the correlation matrix, adjusted PR (APR) along with 95% CIs was executed to determine the association of main independent variables with mothers' and fathers' intentions, and a *p*-value < 0.05 was considered statistically associated. The log-likelihood ratio test and Akaike's and Bayesian information criterion were used to select the final model. The Pearson Chi-square and Hosmer-Lemeshow goodness-of-fit tests were used to test for model integrity.

## Results

### Socio-demographic characteristics of the parents

A total of 1,718 parents of unmarried adolescent girls were identified and included in this study with a response rate of 100%. Of these, 859 were fathers and 859 were mothers. The mean age of the fathers was 43.9 (±7.6) and for the mothers, 38.4 (±6.6) within the range of 28–75 years and 23–60 years for fathers and mothers respectively. Most of the study participants were Muslims (fathers 813; 94.6%, mothers 802; 93.4%), ethnically Oromo (fathers 795; 92.6%, mothers 804; 93.6%), and rural residents (656, 76.19%). Regarding educational status, about 513 (59.7%) of fathers and 498 (57.9%) of mothers were illiterate. Moreover, about 638 (37.1%) of the participants were from poor households (Table 1).

**Table 1:** Background characteristics of parents of unmarried girls living in Kersa HDSS, Eastern Ethiopia, 2019.

**Characteristics**	**Categories**	**Mother (*****n** =* **859)**		**Father (*****n** =* **859)**	
		***n*** **(%)**		***n*** **(%)**	
Age	25–34 years	202	23.52	42	4.89
	35–44 years	492	57.28	454	52.85
	≥45 years	165	19.21	363	42.26
Residence	Rural	656	76.37	656	76.37
	Urban	203	23.63	203	23.63
Childhood residence	Rural	736	85.68	750	87.31
	Urban	123	14.32	109	12.69
Religion	Muslim	813	94.64	802	93.36
	Other	46	5.36	57	6.64
Ethnicity	Other	55	6.40	64	7.45
	Oromo	804	93.60	795	92.55
Educational status	No education	498	57.97	513	59.72
	Primary	264	30.73	205	23.86
	Secondary and above	97	11.29	141	16.41
Occupation	Housewife	776	90.34	—	
	Other	83		129	
	Farmer	—	9.66	730	
Family size	3–5	222	25.84	222	25.84
	6–9	518	60.30	518	60.30
	10 and above	119	13.85	119	13.85
	High	256	29.84	256	29.84
	Medium	284	33.10	284	33.10
Wealth status	Poor	318	37.06	318	37.06

“Other occupation”: merchant, government employee, petty trader, daily laborer.

### Background characteristics of parents with their early marriage intention

In this study, 313 (36.44%) mothers and 272 (31.66%) fathers did not know the legal age of marriage among which 142 (45.37%) mothers and 151 (55.5.3%) fathers had the intention to early marriage of daughters. About 782 (91.04%) mothers and 801 (93.3%) fathers knew at least one health consequence of early marriage, among which more than half of the mothers 408 (52.17%) and 438 (54.68%) fathers mentioned “prolonged labor” as a health consequence. Furthermore, among 222 mothers and 177 fathers who did not know the legal consequences of early marriage, 49.15 and 40.09% of mothers and fathers had an intention of early marriage respectively. Among the respondents who knew the appropriate age of marriage, and the health and legal consequences of early marriage, their source of information was health extension workers (312, 38.7%) for mothers, and radio (304, 38.0%) for fathers. More than half of the mothers (556, 64.7%) and fathers (506, 58.9%) who do not follow any mass media regularly, 227 (40.8%) of mothers and 228 (45.06%) of fathers intended to marry off their daughters early (Table 2).

**Table 2:** Background characteristics of parents with their early marriage intention in Kersa HDSS, Eastern Ethiopia, 2019.

		**Number (%) of parent intention to marry off their daughter early (<18 years)**			
		**Agree**		**Not agree**	
**Characteristics**	**Categories**	**Mother**	**Father**	**Mother**	**Father**
		**341 (39.70 %)**	**374 (43.54%)**	**518 (60.30%)**	**485 (56.46 %)**
Age	25–34 years	77 (38.12%)	19 (45.24%)	125 (61.88%)	23 (54.76%)
	35–44 years	206 (41.87%)	196 (43.17%)	286 (58.13%)	258 (56.83%)
	≥45 years	58 (35.15%)	159 (43.80%)	107 (64.85%)	204 (56.20%)
Residence	Urban	37 (18.23%)	50 (24.63%)	166 (81.77%)	153(75.37%)
	Rural	304(46.34%)	324(49.39%)	352 (53.66%)	332(50.61%)
Educational status	Secondary & above	18 (18.56%)	32 (22.70%)	79 (81.44%)	109 (77.30%)
	Primary	100 (37.88%)	56 (27.32%)	164 (62.12%)	149 (72.68%)
	No education	273 (44.78%)	286 (55.75%)	275 (55.22%)	227 (44.25%)
Knowledge of legal age of marriage	Yes	199 (36.45%)	223 (37.99%)	347 (63.55%)	364 (62.01%)
	No	142 (45.37%)	151 (55.51%)	171 (54.63%)	121 (44.49%)
Knowledge of the health consequences of early marriage	Yes	305 (39.00%)	337 (42.07%)	477 (61.00%)	464 (57.93%)
	No	36 (46.75%)	37 (63.79%)	41 (53.25%)	21 (36.21%)
	Prolonged labor	164 (40.20%)	190 (43.38%)	244 (59.80%)	248(56.62%)
	Excessive bleeding after delivery	72 (43.37%)	58 (46.77%)	94 (56.63%)	66 (53.23%)
	Death of the mother	38 (30.40%)	66 (40.74%)	87 (69.60%)	96 (59.26%)
The health consequences of early marriage	Other	34 (40.96%)	21 (27.27%)	49 (59.04%)	56(72.73%)
Knowledge of the legal consequence of early marriage	Yes	252 (39.56%)	287 (42.08%)	385 (60.44%)	395 (57.92%)
	No	89 (40.09%)	87 (49.15%)	133 (59.91%)	90 (50.85%)
Type of legal consequence	Imprisonment	160 (43.72%)	198 (43.42%)	206 (56.28%)	258 (56.58%)
	Cush payment	63 (30.00%)	72 (36.55%)	147 (70.00%)	125 (63.45%)
	Both	28 (45.90%)	15 (51.72%)	33 (54.10%)	14(48.28%)
Source of information for the health and legal consequences	Health extension	118 (37.82%)	116 (46.59%)	194 (62.18%)	133(53.41%)
	Afosha	117 (38.74%)	50(32.68%)	185(61.26%)	103 (67.32%)
	Radio	51 (44.35%)	136(44.74%)	64 (55.65%)	168 (55.26%)
	Friends	32(41.03%)	41(43.16%)	46(58.97%)	54(56.84%)
Regular mass media follow	None	227 (40.83%)	228 (45.06%)	329 (59.17%)	278 (54.94%)
	Radio	72 (36.00%)	113 (50.00%)	128 (64.00%)	113(50.00%)
	Television	42 (40.78%)	33 (25.98%)	61 (59.22%)	94 (74.02%)

“Other the health Consequences”: Fistula and death of the newborn.

### Personal attitude and social expectations (social norms) of the parents

The prevalence of intention to marry off their daughter early in this study was 341 (39.70%; 95% CI = 36.4–43.0%) among mothers and 374 (43.54%; 95%CI = 40.2–47.1%) among fathers of adolescent girls [chi2(1) = 12.3142 *P* < 0.000]. More than half of the mothers 494 (57.5%), and fathers 487 (56.7%) had a favorable attitude toward early marriage. About 528 (61.5%) of mothers and 577 (67.2%) of fathers agreed on the presence of empirical expectation and 518 (60.3%) of mothers and 543 (63.2%) of fathers agreed on the presence of normative expectation. Moreover, about 663 (77.1%) of mothers and 683 (79.5%) of fathers acknowledged the presence of sanctions against those not favoring early marriage practices in the community (Table 3 and Supplementary Table 1).

**Table 3:** Parents' attitude and social expectations (social norms) in Kersa HDSS, Eastern Ethiopia, 2019.

**Characteristics**	**Categories**	**Mother**		**Father**	
		***n***	**(%)**	***n***	**(%)**
Attitude toward early marriage	Favorable	494	57.51	487	56.69
	Unfavorable	365	42.49	372	43.31
Agreed on presence of empirical expectation	Agreed	528	61.47	577	67.17
	Not agreed	331	38.53	282	32.83
Agreed on presence of normative expectation	Agreed	518	60.30	543	63.21
	Not agreed	341	39.70	316	36.79
Presence of sanctions toward early marriage	Agreed	663	77.18	683	79.51
	Not agreed	196	22.82	176	20.49
Reference group for marriage related issues	Spouse	294	34.23	540	62.86
	Friend	320	37.25	131	15.25
	Relatives	245	28.52	188	21.89
Early marriage is typical/commonly practiced among my reference group	Agreed	639	74.39	705	82.07
	Not agreed	220	25.61	154	17.93
Final decision maker on marriage	Father	497	57.86	497	57.86
	Mother	176	20.49	176	20.49
	Grand parents	107	12.46	107	12.46
	Elder Brother	79	9.20	79	9.20
Intention to marry off < 18 years daughter	Yes	341	39.70	374	43.54
	No	518	60.30	485	56.46

### Factors associated with intention of parents

After adjusted analysis, the prevalence ratio of intention to marry off their daughters early was 2.06 times higher among mothers of rural residents (APR = 2.06; 95% CI: 1.53–2.77), and 1.61 times higher among fathers of rural residents (APR = 1.61; 95% CI: 1.28–2.02) compared to their urban counterparts. The prevalence of intention to marry off their daughter early was lower by 59% (APR = 0.41; 95% CI: 0.27–0.62) among mothers, and lower by 50 % (APR = 0.50; 95% CI: 0.38–0.66) among fathers who had completed secondary education and above, compared to uneducated parents. Similarly, the prevalence ratio of intention to marry off their daughters early was 1.29 (APR = 1.29;95% CI: 1.06–1.57) times higher among mothers, and 2.01(APR = 2.01;95% CI: 1.68–2.43) times higher among fathers who had favorable attitude toward early marriage. About 2.21 (APR = 2.21;95% CI: 1.73–2.79) times more mothers and 1.43 (APR = 1.43;95% CI: 1.19–1.72) times more fathers agreed on the presence of empirical expectations; 1.27 (APR = 1.27;95% CI: 1.07–1.51) times more mothers and 1.32 (APR = 1.32;95% CI: 1.12–1.55) times more fathers agreed on the presence of normative expectations of early marriage in the community compared to their counterparts. Moreover, the prevalence of fear of sanctions, if they did not marry off their daughters early, was high among both mothers (77.18%) and fathers (79.51%), but it was not significantly associated with intention (Table 4).

**Table 4:** Factors associated with intention of parents to early marriage living in Kersa HDSS, Eastern Ethiopia, 2019.

**Variable**	**Parent intention**					
	**Mother**			**Father**		
	**Yes**	**No**	**APR**	**Yes 374**	**No**	**APR**
	**341 (39.70%)**	**518 (60.30 %)**	**(95% CI)**	**374 (43.54%)**	**485 (56.46%)**	**(95% CI)**
**Age**						
25–34 years	77	125	1	19	23	1
35–44 years	206	286	1.02 (0.86–1.23)	196	258	0.88 (0.66–1.17)
≥45 years	58	107	0.91 (0.71–1.16)	159	204	0.89 (0.66–1.18)
**Residence**						
Urban	37	166	1	50	153	1
Rural	304	352	2.06 (1.53–2.77)^***^	324	332	1.61 (1.28–2.02)^***^
**Educational status**						
Secondary and above	18	79	1	32	109	1
Primary	100	164	2.41 (1.60–3.64)^***^	56	149	1.14 (0.82–1.61)
No education	223	275	2.23 (1.46–3.40)^***^	286	227	1.99 (1.50–2.65)^***^
**Knowledge of legal age of marriage**						
Yes	199	347	1	223	364	1
No	142	171	1.09 (0.94–1.27)	151	121	1.29 (1.12–1.48)^***^
**Knowledge of the health consequences**						
Yes	305	477	1	337	464	1
No	36	41	1.1 (0.86–1.40)	37	21	1.24 (1.03–1.50)^**^
**Attitude toward early marriage**						
Unfavorable	98	267	1	89	283	1
Favorable	243	251	1.29 (1.06–1.57)^***^	285	202	2.01 (1.68–2.43)^***^
**Agreed to empirical expectations**						
Not agreed	67	264	1	83	199	1
Agreed	274	254	2.21 (1.73–2.79)^***^	291	286	1.43(1.19–1.72)^***^
**Agreed to normative expectation**						
Not agreed	104	237	1	98	218	1
Agreed	237	281	1.27 (1.07–1.51)^***^	276	267	1.32 (1.12–1.55)^***^
**Presence of sanction**						
Not agreed	58	138	1	54	122	1
Agreed	283	380	1.17 (0.94–1.46)	320	363	1.17 (0.94–1.46)
**Early marriage is highly practiced among reference group**						
Not agreed	66	154	1	43	111	1
Agreed	275	364	1.04 (0.85–1.29)	331	374	1.25 (1.00–1.55)^**^

*** p < 0.01,

** p < 0.05.

## Discussion

This study identified the parents' intention and determinants toward early marriage in eastern Ethiopia using social norms measurement tools and the Theory of Planned Behavior. The prevalence of intention to marry off their daughters early among mothers of unmarried adolescent girls was 39.70 and 43.54% among fathers. Factors such as rural residency, illiteracy, favoring early marriage, and acknowledgment of the presence of empirical and normative expectations to conform to social norms were significantly associated with the parents' intention to marry off their daughters.

The immediate antecedent of behavior, in the Theory of Planned Behavior, is the intention to perform the behavior of interest; the stronger the intention, the more likely the behavior to follow (38). This is supported by previous studies that the intention for early marriage has a significant relationship to early marriage behavior (18).

This study revealed that the majority of the parents (91.04% of mothers and 93.30% of fathers) knew the health consequences, and (74.2% of mothers and 79.4% of fathers) were aware of the legal consequence of early marriage. Regardless, nearly 40% of mothers and 44% of fathers of unmarried adolescent girls had the intention to marry off their daughters early. In agreement with our study, various quantitative and qualitative studies have shown the persistence of support of early marriage by parents of unmarried adolescent girls despite awareness of its negative consequences (42–44). Parents play a significant role in early marriage practices by encouraging their daughters to get married early, sometimes with genuine interest to protect their daughters from a condition that might leave them unmarriageable but also resolve their financial problems (45). Other reasons include protecting family honor, preventing sexual assaults and harassment (18), and conforming to socially acceptable norms (46). As a result, many girls are at higher risk of being married before their eighteenth birthday.

In this study, the prevalence of intention to marry off their daughters early was lower among parents who completed at least secondary school compared to those who had no education. This finding is in line with previous studies showing early marriage preference to be high among the illiterate and those with a primary level of education than secondary or higher levels of education (42, 47, 48). The more educated the parents were, the higher the likelihood of them challenging the norms of early marriage. This is due to education clearly being one of the factors that contribute to changing social and traditional norms toward early marriage (49).

This study showed that the prevalence ratio of intention to marry off their daughters early was about two times higher among mothers and 1.61 times higher among fathers based in rural areas compared to urban spaces. As previous studies showed, parents living in rural areas are more inclined to early marriage than those living in urban areas (47, 50). The influence of social norms is high in rural areas (51) where the parents are likely to have limited awareness and knowledge of the negative health consequences of early marriages (48).

More than half of the parents in this study (57.5% of mothers and 56.7% of fathers) had a favorable attitude toward early marriage. The direct determinant of behavioral intentions as explained in the TPB is the attitude toward the behavior. There is a positive relationship and influence on the intention by controlling someone's behavior to adopt a targeted behavior such as early marriage (38, 52).

This study demonstrated that 61.5% of mothers and 67.2% of fathers agreed on the presence of empirical expectation, 60.3% of mothers and 63.2% of fathers agreed on normative expectation, and 77.2% of mothers and 79.5% of fathers agreed with the presence of sanction toward early marriage practices. This is in agreement with previous findings (18, 32). Moreover, the prevalence ratio of intention to marry off their daughters early was higher among mothers and fathers who agreed on empirical expectations toward early marriage, and among mothers and fathers who agreed on normative expectations compared to their counterparts. This shows early marriage is a social norm in this community.

The present study also found that 57.9% of final decision-makers on issues related to marriage were the fathers of adolescent girls. This finding is similar to various studies that show fathers to be the final decision-makers regarding the marriage of their underage daughters (44, 53, 54). Moreover, as indicated above, the intention to marry off their daughters early was higher among fathers compared to mothers and this reveals that more girls are at risk of early marriage due to the preponderance of the fathers' decision.

Several studies in Ethiopia across various religions and geographical regions confirmed our findings: results from Northwest Ethiopia (99.8% of respondents were orthodox Christians) and Gorche, Southern Ethiopia (the majority of respondents were Protestant) confirmed that the main reasons for parents' support of early marriage practices were perceived social and economic benefits, as well as a strong social stigma attached to girls who remain unmarried after the socially appropriate marriage age of 15 (55). Parents in the East Gojjam Zone, North Region, continue to insist on marrying off their daughters in their mid-teens due to social sanctions associated with delayed marriage (56). Early marriage has also been a conditional preference because adolescent girls and their parents in West Hararghe Ethiopia (the majority of the respondents were Muslims) adhere to local social norms (57).

Studies indicating strong early marriage norms have revealed participants affirming the presence of high levels of both empirical and normative understanding that there would be sanctions when social norms are not complied with (46, 58). This indicates that marital-related social norms sustain the practice of early marriage (54).

Even though early marriage remains prevalent in our study community, the majority of parents do not intend to marry off their daughters before the age of 18. This is the result of ongoing efforts by several stockholders in initiatives such as increased investments in girls' education, increased awareness of the legal marriage age (18 years), and community efforts to highlight the health and economic risks of early marriage and childbearing. In addition, attitudes and expectations toward early marriage have shifted across generations. There is also evidence that convincing traditional and religious leaders to condemn the practice of early marriage and set an example by not marrying off their daughters early plays an important role in shifting trends (59).

Several policies, strategies, and programs have been implemented in Ethiopia over the last several decades to address issues of early marriage, either directly or indirectly (60). This includes the incorporation of women's rights and child protection into the Ethiopian constitution in 1994, as well as the creation of the National Policy on Ethiopian Women in 1993, to establish equitable and gender-sensitive public policies (61–63). The minimum age for marriage was raised from 15 to 18 years old in the revised Family Code of 2000 and the Criminal Code of 2005 (63). To address the high prevalence of early marriage, particularly in rural areas, the National Strategy and Action Plan on Harmful Traditional Practices Against Women and Children was adopted in 2013 (60, 64). The Ministry of Women, Children, and Youth developed a comprehensive National Costed Roadmap in 2019 that included federal and regional plans to end the practice by 2025 (65). Marriage under the age of 18 is now illegal in Ethiopia, according to the country's revised family code (66). However, this is not always enforced in rural Ethiopia.

The strength of this study is that the issue has been looked at from both parents' perspectives, given that women generally appear to be subordinate to their husbands and their viewpoints have been considered.

A limitation of the study is the introduction of social desirability bias attached to face-to-face interviews on a culturally sensitive topic that could have led to underreporting of early marriage intentions. Similarly, some respondents might have agreed or disagreed with the statements on perceived norms and intention in a socially acceptable manner rather than the actual beliefs. However, we made efforts to ensure the privacy of respondents during the interview and match the interviewer and respondent by age and sex to minimize unavoidable biases.

## Conclusion

In this study, though the majority of mothers and fathers know the legal age of marriage, negative health, and legal consequences, about 40% of mothers and 44% of fathers intend to marry off their daughters early. Their intention was associated with parents' favorable attitude toward early marriage and perceived norms (empirical and normative expectations) indicating the prevalence of strong social norms in the community that enforces early marriage intentions. Despite the presence of social norms favoring early marriage, factors such as residing in urban areas and parents with higher educational levels were associated with a reduced likelihood of intention to marry daughters off early. Interventions to reduce intention for early marriage need to address local social norms that enforce it.

## Data availability statement

The raw data supporting the conclusions of this article will be made available by the authors, without undue reservation.

## Ethics statement

The studies involving human participants were reviewed and approved by Institutional Health Research Ethics Review Committee (IHRERC) of Haramaya University Ethiopia with Approval Number (IHRERC/177/2018). The patients/participants provided their written informed consent to participate in this study.

## Author contributions

All authors made substantial contributions to the conception and design of the study, acquisition of data, or analysis and interpretation of data, took part in drafting the article or revising it critically for important intellectual content, and gave final approval of the version to be published.

## Funding

This work was supported by institutional funds from Haramaya University Women Grant with a research code of HUWG-2017-02-04-01 as a PhD student's support. The funders had no role in study design, data collection and analysis, decision to publish, and preparation of the manuscript.

## Conflict of interest

The authors declare that the research was conducted in the absence of any commercial or financial relationships that could be construed as a potential conflict of interest.

## Publisher's note

All claims expressed in this article are solely those of the authors and do not necessarily represent those of their affiliated organizations, or those of the publisher, the editors and the reviewers. Any product that may be evaluated in this article, or claim that may be made by its manufacturer, is not guaranteed or endorsed by the publisher.
