# Supplementary material for: Parents' intention toward early marriage of their adolescent girls in eastern Ethiopia: A community-based cross-sectional study from a social norms perspective
Source: Front Glob Womens Health. 2022 Oct 5;3:911648. doi: 10.3389/fgwh.2022.911648 (PMC9581299; doi:10.3389/fgwh.2022.911648)
Supplement: Supplementary file 1 [file Table_1.DOCX]

**Annex I**

**S1Table.1 Personal attitude, social expectations (social norms), and reference groups related questions**

**To what extent do you agree with the following statements?**

| **Characteristics** | **Response categories** | | **Response** | | | | | | | | |
| --- | --- | --- | --- | --- | --- | --- | --- | --- | --- | --- | --- |
|  |  |  | **Mother** | | | | | | **Father** | | |
| **Attitude towards early marriage** |  | | **n** | | **%** | | **Mean** | | **n** | **%** | **Mean** |
| I think girls should get married before age 18 | 1.Disagree  2.Somewhat disagree  3.Somewhat agree  4.Agree | | 320 185  293  61 | | 37.25  21.54  34.11  7.10 | | 2.1 | | 236  162  297  164 | 27.47  18.86  34.58  19.09 | 2.5 |
| In my opinion getting married before 18 years is beneficial | 1.Disagree  2.Somewhat disagree  3.Somewhat agree  4.Agree | | 324 178 284 73 | | 37.72  20.72  33.06  8.50 | | 2.1 | | 239  126  289  205 | 27.82  14.67  33.64  23.86 | 2.5 |
| If a girl did not marry early, she would not be marriageable | 1.Disagree  2.Somewhat disagree  3.Somewhat agree  4.Agree | | 353  236  231 39 | | 41.09  27.47  26.89  4.54 | | 1.9 | | 296  148  264  151 | 34.46  17.23  30.73  17.58 | 2.3 |
| An older girl will not find a good husband | 1.Disagree  2.Somewhat disagree  3.Somewhat agree  4.Agree | | 311  55  265  228 | | 36.20  6.40  30.85  26.54 | | 2.5 | | 227  156  324  152 | 26.43  18.16  37.72  17.69 | 2.5 |
| **Attitude towards early marriage**  **Positive Attitude**  **Negative Attitude** | | | **494**  **365** | | **57.51**  **42.49** | | **8.7** | | **487**  **372** | **56.69**  **43.31** | **9.7** |
| **Presence of empirical expectation** | | | | | | | | | | | |
| Most of girls in my community marry before the age of 18. | 1.Disagree  2.Somewhat disagree  3.Somewhat agree  4.Agree | | 93  141 321 304 | | 10.83  16.41  37.37  35.39 | | | 3.0 | 77 75 168 539 | 8.96  8.73  19.56  62.75 | 3.4 |
| Most parents/(mothers and fathers) in this community marry off their daughter early (<18) | 1.Disagree  2.Somewhat disagree  3.Somewhat agree  4.Agree | | 105  135  274  345 | | 12.22  15.72  31.90  40.16 | | | 3 | 58  136  97 568 | 6.75  15.83  11.29  66.12 | 3.4 |
| All my neighbors marry off their daughter’s as soon as they reach puberty. | 1.Disagree  2.Somewhat disagree  3.Somewhat agree  4.Agree | | 113  158  270  318 | | 13.15  18.39  31.43  37.02 | | | 2.9 | 93  121  102  543 | 10.83  14.09  11.87  63.21 | 3.3 |
| Parents (mothers and fathers)marry off their daughters early (<18years) because they believe other parents also do the same. | 1.Disagree  2.Somewhat disagree  3.Somewhat agree  4.Agree | | 134 182  308  235 | | 15.60  21.19  35.86  27.36 | | | 2.7 | 72  100  138  549 | 8.38  11.64  16.07  63.91 | 3.4 |
| **Presence of empirical expectation**  **Agreed**  **Not agreed** | | | **528**  **331** | | **61.47**  **38.53** | | | **11.6** | **577**  **282** | **67.17**  **32.83** | **13.3** |
| **Presence of normative expectation** | | | | | | | |  |  |  |  |
| Most of my friends think that girls should get married before age 18 | | 1.Disagree  2.Somewhat disagree  3.Somewhat agree  4.Agree | | 156 209  352  142 | | 18.16  24.33  40.98  16.53 | | 2.6 | 136 158  259  306 | 15.83  18.39  30.15  35.62 | 2.9 |
| Parents/father and mother/ expect adolescent girls to get married before the age of 18 years | | 1.Disagree  2.Somewhat disagree  3.Somewhat agree  4.Agree | | 133  173 310 243 | | 15.48  20.14  36.09  28.29 | | 2.8 | 175 126 198  360 | 20.37  14.67  23.05  41.91 | 2.8 |
| Community elders expect parents marry off their girls before the age of 18 years. | | 1.Disagree  2.Somewhat disagree  3.Somewhat agree  4.Agree | | 130 163  319 247 | | 15.13  18.98  37.14  28.75 | | 2.8 | 187 139  207 326 | 21.77  16.18  24.10  37.95 | 2.8 |
| Religious leaders expect parents marry off their girls before the age of 18 years. | | 1.Disagree  2.Somewhat disagree  3.Somewhat agree  4.Agree | | 134 171  308  246 | | 15.60  19.91  35.86  28.64 | | 2.8 | 149 155  196 359 | 17.35  18.04  22.82  41.79 | 2.9 |
| Others community members expect parents marry off their daughters before the age of 18 years | | 1.Disagree  2.Somewhat disagree  3.Somewhat agree  4.Agree | | 91  190  297  281 | | 10.59  22.12  34.58  32.71 | | 2.9 | 109  195  189  366 | 12.69  22.70  22.00  42.61 | 2.9 |
| **Presence of normative expectation**  Agreed  Not agreed | | | | **518**  **341** | | **60.30**  **39.70** | | **13.8** | **543**  **316** | **63.21**  **36.79** | **14.3** |
| **Sanctions** | | | | | | | | | | | |
| Parents would look down on adolescent girls if they get pregnant before they get married | | 1.Disagree  2.Somewhat disagree  3.Somewhat agree  4.Agree | | 19  70  199 571 | | 2.21  8.15  23.17  66.47 | | 3.5 | 25  39 177 618 | 2.91  4.54  20.61  71.94 | 3.6 |
| Parents who marry off their daughters early(<18) marked in the community as good parenthood | | 1.Disagree  2.Somewhat disagree  3.Somewhat agree  4.Agree | | 9  39  254  557 | | 1.05  4.54  29.57  64.84 | | 3.6 | 24  39 223  573 | 2.79  4.54  25.96  66.71 | 3.6 |
| **Presence of sanctions**  Agreed  Not agreed | | | | **663**  **196** | | **77.18**  **22.82** | | **7.1** | **683**  **176** | **79.51**  **20.49** | **7.2** |
| **Early marriage reference group** | | | | | | | | | | | |
| I believe that early marriage is commonly practiced by them (typical among my reference group)? | | 1.Disagree  2.Somewhat disagree  3.Somewhat agree  4.Agree | | 125  76 313  270 | | 15.94  9.69  39.92  34.44 | | 2.9 | 107  47  411  294 | 12.46  5.47  47.85  34.23 | 3.0 |
| **Early marriage is typical among my reference group**  Agreed  Not agreed | | | | **639**  **220** | | **74.39**  **25.61** | | **2.9** | **705**  **154** | **82.07**  **17.93** | **3.0** |
